# Fast sequence evolution of *Hox *and *Hox*-derived genes in the genus *Drosophila*

**DOI:** 10.1186/1471-2148-6-106

**Published:** 2006-12-12

**Authors:** Sònia Casillas, Bárbara Negre, Antonio Barbadilla, Alfredo Ruiz

**Affiliations:** 1 Departament de Genètica i de Microbiologia, Universitat Autònoma de Barcelona, 08193 Bellaterra (Barcelona), Spain; 2 Department of Zoology, University of Cambridge, Cambridge CB2 3EJ, UK

## Abstract

**Background:**

It is expected that genes that are expressed early in development and have a complex expression pattern are under strong purifying selection and thus evolve slowly. *Hox *genes fulfill these criteria and thus, should have a low evolutionary rate. However, some observations point to a completely different scenario. *Hox *genes are usually highly conserved inside the homeobox, but very variable outside it.

**Results:**

We have measured the rates of nucleotide divergence and indel fixation of three *Hox *genes, *labial *(*lab*), *proboscipedia *(*pb*) and *abdominal-A *(*abd-A*), and compared them with those of three genes derived by duplication from *Hox3*, *bicoid *(*bcd*), *zerknüllt *(*zen*) and *zerknüllt-related *(*zen2*), and 15 non-*Hox *genes in sets of orthologous sequences of three species of the genus *Drosophila*. These rates were compared to test the hypothesis that *Hox *genes evolve slowly. Our results show that the evolutionary rate of *Hox *genes is higher than that of non-*Hox *genes when both amino acid differences and indels are taken into account: 43.39% of the amino acid sequence is altered in *Hox *genes, versus 30.97% in non-*Hox *genes and 64.73% in *Hox*-derived genes. Microsatellites scattered along the coding sequence of *Hox *genes explain partially, but not fully, their fast sequence evolution.

**Conclusion:**

These results show that *Hox *genes have a higher evolutionary dynamics than other developmental genes, and emphasize the need to take into account indels in addition to nucleotide substitutions in order to accurately estimate evolutionary rates.

## Background

*Hox *genes are homeobox containing genes involved in the specification of regional identities along the anteroposterior body axis and, thus, play a fundamental role in animal development [1]. They encode transcription factors that regulate the expression of other genes downstream in the regulatory cascade of development and have been found in all metazoans, including flies, worms, tunicates, lampreys, fish and tetrapods. A particular feature of these genes is that they are usually clustered together in complexes and arranged in the chromosome in the same order as they are expressed along the anteroposterior body axis of the embryo [2,3]. Ten genes arranged in a single complex comprised the ancestral *Hox *gene complex of arthropods (HOM-C) [4-6]. However, at least three different HOM-C splits have occurred during the evolution of diptera [7-10], and several non-homeotic genes and other genes derived from ancestral *Hox *genes are interspersed among the *Drosophila Hox *genes.

The stability of *Hox *gene number and the conservation of *Hox *ortholog sequences prompted the notion that *Hox *proteins have not significantly diverged in function. However, it is now known that several arthropod *Hox *proteins have changed in sequence and/or function, including those encoded by *Hox3 *[11-13], *fushi tarazu *(*ftz*) [14], *Ultrabithorax *(*Ubx*) [15] and *Antennapedia *(*Antp*) [16]. In winged insects, including *Drosophila*, *Hox3 *and *ftz *lost their homeotic function, that is, their ability to transform the characteristics of one body part into those of another body part [17,18], and their expression domains are no longer arranged along the anteroposterior axis of the embryo. Therefore, only eight *Hox *genes remain in these species [6]. *Hox3 *gained a novel extraembryonic function, and underwent two consecutive duplications that gave rise to *bicoid *(*bcd*), *zerknüllt *(*zen*) and *zerknüllt-related *(*zen2*). The first duplication took place in the cyclorrhaphan fly lineage and gave rise to *zen *and *bcd *[12,13]. Afterwards, but before the *Drosophila *radiation, *zen *went through a second duplication that gave birth to *zen2 *[19]. Seemingly, *bcd *and *zen *have specialized and perform separate functions in the establishment of the embryo's body plan: the maternal gene *bcd *codes for an important morphogen that establishes anteroposterior polarity [20] and *zen *is a zygotic gene involved in dorsoventral differentiation [21]. *zen2 *has the same expression pattern of *zen*, although its function is unknown. Despite its high sequence divergence across species, it has been maintained for more than 60 Myr [19].

*Hox *proteins contain a highly conserved domain of 60 amino acids (coded by the homeobox) that binds DNA through a '*helix-turn-helix*' structure. This motif is very similar in terms of sequence and structure to that of many DNA binding proteins. Functional comparisons of *Hox *orthologs have largely focused on their highly conserved homeodomain sequences and have demonstrated their functional interchangeability between species [22-26]. *Hox*-derived genes, although having lost their homeotic function, still retain the homeobox.

It has been shown that housekeeping genes, which are expressed in all cells and at all times, are under strong purifying selection and thus evolve slowly (e.g. histones, or genes involved in the cell cycle) [27,28]. *Hox *genes, on the contrary, are expressed early in development and have a complex regulated expression pattern. Mutations in such genes will on average have more deleterious fitness consequences than mutations occurring in genes expressed later on, because they may have cascading consequences for the later steps in development and thus may broadly alter the adult phenotype [29-31]. Therefore, we also expect *Hox *genes to be highly constrained and thus evolve slowly. In fact, Davis, Brandman, and Petrov [29] found a highly significant relationship between the developmental timing of gene expression and their nonsynonymous evolutionary rate: genes expressed early in development are likely to have a slower rate of evolution at the protein level than those expressed later. Surprisingly, the strongest negative relationship between expression and evolutionary rate occurred only after the main burst of expression of segment polarity and *Hox *genes in embryonic development, so these genes could be evolving differently from other developmental genes. However, only one segment polarity gene, *wingless *(*wg*), and two *Hox *genes, *Antp *and *abdominal-A *(*abd-A*), were analyzed.

Furthermore, Marais *et al*. [32] found a negative correlation between evolutionary rate at the protein level (as measured by the number of nonsynonymous substitutions per nonsynonymous site, *d*_*N*_) and intron size in *Drosophila*, likely due to a higher abundance of *cis*-regulatory elements in introns (especially first introns) in genes under strong selective constraints. We know from a previous study that the *Hox *genes used in this study contain a long intron replete with regulatory elements [19]. Therefore, we would expect these genes to be strongly constrained.

However, other studies seem to point to a completely different scenario. Developmental biologists noticed a long time ago that a large portion of the sequence of *Hox *proteins diverges so fast that it is difficult to align homologues from different arthropod classes [33]. In fact, nucleotide sequences outside the homeobox in *labial (lab) *and *Ubx *have been reported to diverge significantly [8,15]. These sequence differences may be neutral with respect to protein function or, more intriguingly, they could be involved in the functional divergence of *Hox *proteins and the evolutionary diversification of animals [15]. Moreover, Karlin and Burge [34] have shown that many essential developmental genes, including *Hox *genes, contain long microsatellites within their coding sequence (e.g. trinucleotide repeats that do not disrupt the open reading frame). The vast majority of these genes function in development and/or transcription regulation, and are expressed in the nervous system. Due to the particular mutation mechanism acting on these repetitive sequences by replication slippage [35,36], microsatellites are subject to frequent insertions and deletions. Thus, these repetitive sequences could be responsible for a higher than expected evolutionary rate of *Hox *genes. However, and despite all the previous contributions, no quantification of the rates of nucleotide and indel evolution has been reported so far for a set of *Hox *genes.

On the other hand, the origin by duplication and the functional evolution of *Hox*-derived genes suggest that they might be evolving fast at the sequence level as well. Duplicated genes are known to undergo a period of accelerated evolution where: they may degenerate to a pseudogene (pseudogenization), each daughter gene may adopt part of the functions of their parental gene (subfunctionalization), or they may acquire new functions (neofunctionalization) [37-40]. The only divergence estimate reported in a *Hox*-derived gene was calculated between two close species (*D. melanogaster *and *D. simulans*) in *bcd *[41]. A recent study found an increased sequence polymorphism in *bcd *in comparison to *zen*, which was ascribed to a relaxation of selective constraint on this maternal gene resulting from sex-limited expression [42]. Therefore, *bcd *is expected to evolve faster than *zen *under this model. The evolutionary rates of *zen *and *zen2*, however, have not been reported so far.

We have measured the rates of nucleotide substitution and indel fixation of three *Hox *genes, *lab*, *proboscipedia *(*pb*) and *abd-A*, and compared them with those of *bcd*, *zen *and *zen2*, which were derived by duplication from *Hox3*, and a sample of 15 non-*Hox *genes, in the genus *Drosophila*. These rates were compared to test the hypothesis that *Hox *genes, similar to other genes with complex expression patterns and that are essential in the early development, evolve slowly. We have also evaluated the contribution of the homeobox and the repetitive regions within *Hox *and *Hox*-derived genes to the evolutionary rates.

The sequences compared comprise all the complete genes available in *D. buzzatii *(representative of the Drosophila subgenus), and their orthologs in *D. melanogaster *and *D. pseudoobscura *(both species in the Sophophora subgenus). *D. buzzatii *belongs to the *repleta *species group, a group comprising ~100 species that has been widely used as a model in studies of genome evolution, ecological adaptation and speciation. Negre *et al*. [19] have recently compared the genomic organization of the HOM-C complex in *D. buzzatii *to that of *D. melanogaster *and *D. pseudoobscura*, and studied the functional consequences of two HOM-C splits present in this species. When our study began, this was the largest set of orthologous *Hox *genes in species from both subgenera of the *Drosophila *genus, and this allowed the exploration of evolutionary rates throughout the *Drosophila *phylogeny. Due to the high divergence of *Hox *genes [8], the inclusion of more distant species outside the *Drosophila *genus (such as mosquito or honeybee) would probably not be appropriate for the estimation of genetic distances. Moreover, these species do not contain the *Hox*-derived genes studied here.

## Results

### Nucleotide evolution of *Hox*, *Hox*-derived and non-*Hox *genes

Nucleotide substitution parameters were calculated for the coding nucleotide alignments independently for each gene [see Additional file 1]. We then tested for differences between the three groups of genes (*Hox*, *Hox*-derived and non-*Hox*) (top section of Table 1) [see Additional file 2]. Our results showed that *Hox*-derived genes are evolving much faster and with less functional constraint than *Hox *and non-*Hox *genes. Differences among the three groups are significant for the number of nonsynonymous substitutions per nonsynonymous site, *d*_*N *_(P = 0.022), and the level of functional constraint, ω (P = 0.000) (see Methods). The gene *zen2 *is the main gene responsible for the high values of nucleotide substitutions (both synonymous and nonsynonymous) in its group [see Additional file 1]. On the contrary, *Hox *and non-*Hox *genes have a similar number of nucleotide substitutions, *t *(P > 0.1). However the level of functional constraint is even higher (lower ω) in non-*Hox *genes than in *Hox *genes (ω = 0.04156 versus ω = 0.06094, respectively), although differences are only marginally significant (P = 0.063). Therefore, *Hox *genes do not seem to be evolving more slowly than other non-homeotic genes, despite their essential function in early development.

**Table 1:** Mean nucleotide substitution parameters and ANOVAs for the three groups of genes.

		***t***	***d*_*N*_**	***d*_*S*_**	**ω**
Complete coding sequences	*Hox*	2.10917	0.15964	2.59066	0.06094
	*Hox*-derived	3.86336	0.39380	4.27598	0.09226
	Non-*Hox*	2.91160	0.15802	3.80668	0.04156
	
	ANOVA	n.s.	*	n.s.	***

Coding sequences excluding the homeobox	*Hox*	2.27653	0.18257	2.65921	0.06673
	*Hox*-derived	5.04914	0.54809	5.26666	0.11320
	Non-*Hox*	2.91160	0.15802	3.80668	0.04156
	
	ANOVA	n.s.	**	n.s.	***

Coding sequences excluding repetitive regions	*Hox*	1.81997	0.12399	2.35029	0.05310
	*Hox*-derived	3.71981	0.37759	4.14242	0.09042
	Non-*Hox*	2.85593	0.15444	3.76458	0.04035
	
	ANOVA	n.s.	*	n.s.	***

Coding sequences excluding the homeobox and repetitive regions	*Hox*	1.94286	0.14684	2.33783	0.06146
	*Hox*-derived	4.88928	0.53011	5.12014	0.11245
	Non-*Hox*	2.85593	0.15444	3.76458	0.04035
	
	ANOVA	n.s.	**	n.s.	***

n.s. (P>0.05), * (P<0.05), ** (P<0.01), *** (P<0.001)

**Figure 1:**
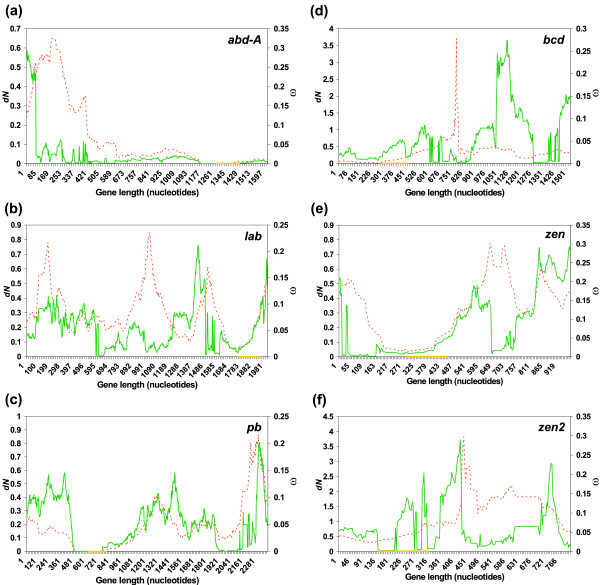
**Distribution of *d*_*N *_and ω in sliding windows along the coding sequence of genes**. Distribution of *d*_*N *_(broken line) and ω (solid line) in sliding windows of 240 nucleotides. (a) *abd-A*, (b) *lab*, (c) *pb*, (d) *bcd*, (e) *zen *and (f) *zen2*. In each case, the position of the homeobox is represented by a yellow box within the X axis.

Then, we plotted *d*_*N *_and ω in sliding windows along the coding sequences of *Hox *and *Hox*-derived genes to see whether or not these parameters behave homogeneously along the sequence. Figure 1 shows that, in all genes except *zen2*, there is a substantial decrease of both *d*_*N *_and ω near the homeobox. *zen2 *contains a rapidly evolving homeobox with high ω values. Contrarily, we have observed that peaks of *d*_*N *_tend to lie within repetitive regions (data not shown).

To control for a possible effect on the overall nucleotide evolution of both the homeobox and the repetitive regions (see Methods) of these *Hox *and *Hox*-derived genes, we tested again for differences among the three groups of genes excluding these regions. Removing the homeobox in *Hox *and *Hox*-derived coding sequences (second section of Table 1) elevated the number of nucleotide substitutions in these two groups, and decreased further their level of functional constraint. Again, differences among groups were significant for *d*_*N *_(P = 0.005) and ω (P = 0.000), and the same tendency of the previous analysis with complete coding sequences was observed. In contrast, removing repetitive regions (third section of Table 1) decreased the number of nucleotide substitutions, especially in *Hox *genes, where all the genes in the group contain this type of region. Therefore, the elimination of repetitive regions slightly increases the difference between *Hox *and non-*Hox *genes in terms of nucleotide substitutions, and reduces the difference in functional constraint. Once more, differences among groups were significant for *d*_*N *_(P = 0.030) and ω (P = 0.001). Excluding both the homeobox and the repetitive regions (bottom section of Table 1) gave intermediate results. Therefore, we can conclude that: (1)*Hox *and non-*Hox *genes are evolving similarly in terms of nucleotide substitutions, (2) *Hox*-derived genes are evolving much faster and with less functional constraint than the other two groups of genes, and (3) neither the homeobox nor the repetitive regions alter the estimates significantly, and thus are not entirely responsible for the two previous conclusions.

An excess of nonsynonymous over synonymous substitutions is a robust indicator of positive selection at the molecular level. Therefore, we searched for values of nonsynonymous/synonymous rate ratio (*d*_*N*_*/d*_*S *_= ω) greater than 1 to investigate whether Darwinian selection has been acting on any of the coding sequences analyzed in this study. However, no evidence of positive selection in any coding sequence or region of it was found.

### Amino acid and structural changes at the protein level

**Figure 2:**
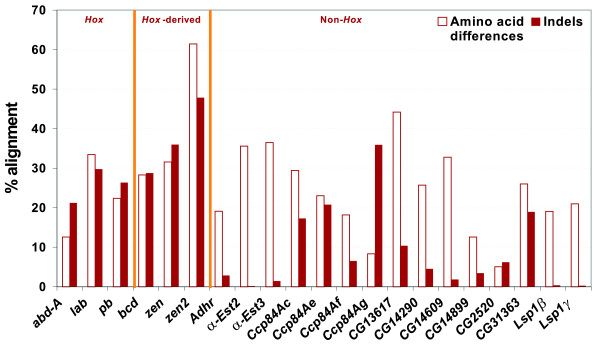
Proportion of amino acid differences and indels in the set of genes analyzed in this study.

We used the protein alignments to calculate the proportion of amino acid differences and indels. In the first case (Table 2, Figure 2), differences among the three groups – *Hox*, *Hox*-derived and non-*Hox *– were not significant (P = 0.101). However, the proportion of amino acid differences was substantially higher for *Hox*-derived genes (40.43%) than for *Hox *and non-*Hox *genes (22.80% and 23.77%, respectively). This result is in full agreement with our previous estimates of *d*_*N *_(Table 1), which showed high values of this parameter for *Hox*-derived genes, but very similar values for *Hox *and non-*Hox *genes.

**Table 2:** Percentage of amino acid differences in the alignment (± SD) in the three groups of proteins.

	**TOTAL**	**UNIQUE**	**REPETITIVE**	**T-test^§^**
***Hox***	22.80 ± *10.44*	18.22 ± *10.50*	37.11 ± *12.33*	
***Hox*-derived**	40.43 ± *18.26*	39.00 ± *19.64*	62.97 ± *24.08*	***
**Non-*Hox***	23.77 ± *10.81*	23.38 ± *10.93*	55.46 ± *31.35*	

**ANOVA**	n.s.	n.s.	n.s.	

n.s. (P > 0.05), * (P < 0.05), ** (P < 0.01), *** (P < 0.001).

^§ ^T-test for paired samples (unique *vs*. repetitive) on proteins having both types of regions [ABD-A, LAB, PB, BCD, ZEN, Ccp84Ac, CG13617, CG14290 and LAP (product of *CG2520*)].

Second, we analyzed the proportion of indels in the alignments (Table 3, Figure 2). In this case, differences among the three groups of genes were highly significant (P = 0.000). Surprisingly, differences were due to the low indel proportion in non-*Hox *genes (8.73%) compared to the high values for *Hox *and *Hox*-derived genes (25.77% and 37.53%, respectively). Furthermore, we tested for differences in indel length using a nested ANOVA. The results indicated that, although the variation in indel length between genes within groups is significant (P = 0.021), the difference between groups is even more significant (P = 0.001). Mean indel length for *Hox*, *Hox*-derived and non-*Hox *genes is 4.22, 5.99 and 3.55 amino acids, respectively. Non-*Hox *genes not only have on average shorter indels, but also their longest indel is only 23 amino acids, in comparison with 43 and 40 amino acids for *Hox *and *Hox*-derived genes, respectively. In all groups, the indel length distribution follows a negative exponential curve: short indels are common and their abundance declines as length increases (data not shown).

**Table 3:** Percentage of indels in the alignment (± SD) in the three groups of proteins.

	**TOTAL**	**UNIQUE**	**REPETITIVE**	**T-test^§^**
***Hox***	25.77 ± *4.31*	16.21 ± *8.40*	44.82 ± *2.38*	
***Hox*-derived**	37.53 ± *9.63*	34.88 ± *12.40*	75.64 ± *34.45*	**
**Non-*Hox***	8.73 ± *10.24*	8.46 ± *10.28*	23.79 ± *25.66*	

**ANOVA**	***	**	n.s.	

n.s. (P > 0.05), * (P < 0.05), ** (P < 0.01), *** (P < 0.001).

^§ ^T-test for paired samples (unique *vs*. repetitive) on proteins having both types of regions [ABD-A, LAB, PB, BCD, ZEN, Ccp84Ac, CG13617, CG14290 and LAP (product of *CG2520*)].

Finally, we tested whether the proportions of amino acid differences and indels are correlated. The Pearson correlation indicated that these two variables are positively but not significantly correlated (r_Pearson _= 0.307, P = 0.175). Therefore, genes with a high proportion of indels do not necessarily have a high proportion of amino acid substitutions. This probably points to different causal mechanisms for amino acid substitutions and indels.

### Effect of long repetitive tracks in the percentages of amino acid differences and indels of *Hox *and *Hox*-derived proteins

**Figure 3:**
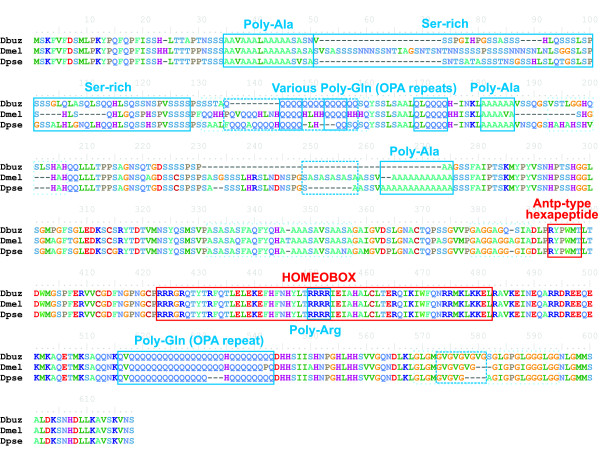
**Alignment of a *Hox *protein (ABD-A) showing multiple long repeats spacing functional domains**. Functional domains are represented by red boxes, and repeats by blue boxes as follows: repetitive regions annotated in UniProt are represented by solid boxes, simple repeats by dashed boxes and complex repeats by dotted light boxes (see Methods). Notation: Dbuz = *D. buzzatii*; Dmel = *D. melanogaster*; Dpse = *D. pseudoobscura*.

Most *Hox *and *Hox*-derived proteins contain large repetitive regions present throughout the protein except the region near the homeobox and other highly conserved regions (see for instance the amino acid sequence of ABD-A in Figure 3). Predominant repetitions are poly-glutamine (poly-Q), poly-alanine (poly-A) and serine-rich regions (S-rich). These repetitive regions seem to include most of the indels and amino acid differences, and therefore they might be responsible for the surprisingly high evolutionary rate of *Hox *and *Hox*-derived proteins.

To test this hypothesis, we repeated the analyses of amino acid differences and indels inside and outside these repetitive regions (see Methods), and compared these two kinds of sequences (repetitive and unique). In the case of amino acid differences (Table 2), the percentage of aligned, non-conserved amino acids is higher in repetitive regions than in unique sequence in all the three groups. The T-test for paired samples (unique versus repetitive) on proteins having both types of regions showed significant differences between unique and repetitive sequences (P = 0.001), the mean of repetitive sequences being more than twice that for unique sequences (51.01% versus 23.19%, respectively). Despite this higher percentage of amino acid differences in repetitive than in unique sequence, the three groups of genes behave in a similar manner in both types of regions (note that the ranking is the same in both unique and repetitive regions).

Finally, we wanted to determine whether or not repetitive regions accumulate a larger number of indels than unique sequence (Table 3). The results show that in all the three groups, the percentage of indels in repetitive regions is much higher than that in unique sequence. These differences are significant (P = 0.006) according to a T-test for paired samples, giving an average value of 42.32% in repetitive regions versus 15.53% in unique sequence. Nevertheless, the ANOVA computed after removing repetitive regions remained highly significant (P = 0.003). Thus repetitive regions are not entirely responsible for the high percentage of indels in *Hox *and *Hox*-derived proteins. Therefore, *Hox *and *Hox*-derived genes have a tendency to accumulate indels even outside of repetitive regions, which does not seem to be allowed in non-*Hox *genes.

## Discussion

### Evolutionary rates of *Hox *genes

This study shows that *Hox *genes seem to be evolving differently from other essential genes expressed in early development, with complex expression patterns or with long introns rich in *cis*-regulatory elements. Both the number of nonsynonymous substitutions and the degree of functional constraint are not significantly different between *Hox *and non-*Hox *genes, and this remains true even when the most peculiar regions (the homeobox and the repetitive regions) are excluded (Table 1). Therefore, *Hox *genes do not seem to be evolving more slowly than other non-homeotic genes, despite their essential function in the early development and even though their interchangeability between species has been proven to be functional in some cases [22-26].

Differences in the evolutionary rate among the three groups of genes (*Hox*, *Hox*-derived and non-*Hox*) could be mediated by some properties of genes that are correlated with the number of nucleotide substitutions (*t*). One possibility is that *Hox *and *Hox*-derived genes experience similar background rates of mutation that are different from those of non-*Hox *genes. We can use the number of synonymous substitutions per synonymous site (*d*_*S*_) as a measure of the mutation rate of a gene. This variable is not significantly different among the three groups of genes (P = 0.530), and thus we can consider that mutation rate is constant across groups [see Additional file 2]. Another possibility is that genes within a group may have correlated levels of synonymous codon bias. Given that genes with higher codon bias tend to evolve more slowly [28,43], codon bias may contribute to spurious differences in the rates of protein evolution among groups. We have measured codon bias for each gene using the Effective Number of Codons, *N*_*C *_[44]. There are no significant differences in the codon bias among groups, and the average *N*_*C *_value for non-*Hox *genes is the lowest among the three groups (the highest codon bias) [see Additional files 1 and 2].

Some *Hox *and *Hox*-derived genes considered here have been included in previous studies [29,41]. Davis *et al*. [29] showed that the strongest negative relationship between expression profile and evolutionary rate occurs at a late stage in embryonic development, soon after the main burst of expression of segment polarity and *Hox *genes. However, they also show that the most constrained transcription factors and signal transducers, the functional class that contains many developmentally essential genes, are expressed precisely at the same time as the segment polarity and *Hox *genes. One of the two *Hox *genes included in their study has also been analyzed here (*abd-A*), and it is incidentally the gene with the lowest number of nonsynonymous substitutions and the one that is most constrained in our sample of *Hox *genes. On the other hand, *bcd*, although being one of the first genes acting in *Drosophila *development, was reported in the same study as an exceptional case of a gene acting in the earliest stages of development but evolving surprisingly fast [29].

Furthermore, *Hox *genes depart from a negative correlation found in previous studies between evolutionary rate at the protein level and intron size, number of conserved noncoding sequences within introns, or regulatory complexity [32]. In this respect, all *Hox *genes used in this study contain a total intron size >10 Kb [see Additional file 3], which corresponds to the longest intron size category used in [32]. Therefore, *Hox *genes are expected to evolve slowly as they contain long intronic sequences. Both *Hox*-derived and non-*Hox *genes contain shorter intron lengths than *Hox *genes [see Additional file 3], and thus would be expected to evolve faster.

### Amino acid differences and indels

The percentages of amino acid differences and of indels in *Hox *proteins also depart from the initial expectations. While the percentage of amino acid differences is not significantly different among the three groups compared (Table 2), the percentages of indels in *Hox *and *Hox*-derived proteins are much higher than that in non-*Hox *proteins (Table 3). Therefore, *Hox *proteins are as divergent as non-*Hox *proteins in terms of amino acid changes, but they are much more divergent in terms of indels. A visual inspection of the alignments pointed out a possible explanation to these results (Figure 3). *Hox *and some *Hox*-derived proteins contain large repetitive regions, mostly homopeptides, present all along the protein except the region near the homeodomain and other highly conserved regions. It is within these repetitive regions where most indels and amino acid differences seem to accumulate, in some cases resulting in poor alignment, and therefore they could be responsible for the surprisingly high amino acid and indel evolution of *Hox *and *Hox*-derived proteins.

Although repetitive regions have been shown to be richer in amino acid differences and indels than unique sequence, they do not fully explain the high variation found in *Hox *and *Hox*-derived proteins. Even excluding repetitive regions, *Hox *and *Hox*-derived genes contain many more indels than non-*Hox *genes, although the percentage of amino acid substitutions is not significantly different between *Hox *and non-*Hox *genes. Therefore, taking amino acid differences and indels altogether we can state that the overall rate of evolution of *Hox *and *Hox*-derived genes is faster than that of non-*Hox *genes. The percentage of the alignment that has changed is 43.39% in *Hox *proteins, 64.73% in *Hox*-derived proteins and 30.97% in non-*Hox *proteins (the percentage of amino acid differences has been recalculated before being added to the percentage of indels to account for the total number of sites, both gapped and non-gapped, in order to make both percentages comparable). Finally, a lack of correlation between the proportion of indels and amino acid differences in the set of genes used in this study highlights the different evolutionary mechanisms that regulate both types of changes.

### Homopeptides and other repetitions in *Hox *and *Hox*-derived proteins

Multiple long homopeptides are found in 7% of *Drosophila *proteins, most of which are essential developmental proteins expressed in the nervous system and involved in transcriptional regulation [34,45]. What is the role of these homopeptides? They could be tolerated, non-essential insertions that may play a role as transcriptional activity modulators. Some examples have been described in *Hox *and *Hox*-derived proteins [15] that illustrate the acquisition of new functions in the insect lineage while maintaining their homeotic role. In these examples, selection against coding changes might have been relaxed because of functional redundancy among *Hox *paralogs. These sequence differences could be involved in the functional divergence of *Hox *proteins and the evolutionary diversification of animals [15].

The large effects of *Hox *genes on morphology suggest that they regulate, directly or indirectly, a large number of genes. It would be expected that such pleiotropic proteins would be constrained in their sequence variation and, hence, their contribution to morphological variation. However, it has been shown that microsatellite sequences in developmental genes are a source of variation in natural populations, affecting visible traits by expanding or contracting at very high rates [46]. One intrinsic characteristic of microsatellites is their hypervariability, resulting from a balance between slippage events and point mutations [35,36]. Their mutation rate has been estimated to be 1.5 × 10^-6 ^per locus per generation in the case of trinucleotide repeats in *D. melanogaster *[47], and is even greater in the case of dinucleotides. These values contrast with the general mutation rate of ~10^-8 ^per site per generation of base pair substitutions [48]. These repeats typically generate regions in the alignment with high variability in sequence and length, and that are difficult to align.

A potential role for homopeptides is to serve as spacer elements between functional domains, to provide flexibility to the three-dimensional conformation, and fine-tuning domain orientation of the protein in its interactions with DNA and other proteins. To that effect, changes in nucleotide distances between target binding sites might be accompanied by complementary changes in the sequences spacing the binding domains of transcription factors (mostly homopeptides). This would produce a coordinated evolution between transcription factors and their target binding sites. Excessive expansions of homopeptides, however, have often been associated with disease in humans [49-52]. Amazingly, essential developmental proteins like homeotic proteins that apparently need such homopeptides for their correct functioning have to suffer the consequences of their quick and apparently unpredictable evolution, and sacrifice in this way the conservation that would be expected in proteins of this type.

Among non-*Hox *genes, the cluster of cuticular genes (*Ccp84Ac*, *Ccp84Ae*, *Ccp84Af *and *Ccp84Ag*) behave similarly to *Hox *and *Hox*-derived genes and account for the vast majority of indels in their group (Figure 2). These short proteins share a conserved C-terminal section [53] and include a 35–36 amino acid motif known as the R&R consensus, present in many insect cuticle proteins, an extended form of which has been shown to bind chitin (chitin-bind 4; PF00379) [54]. Outside these conserved domains, cuticular proteins share hydrophobic regions dominated by tetrapeptide repeats (A-A-P-A/V), which are presumed to be functionally important [55,56] and are responsible for the high percentage of indels found in these proteins. These repeats are usually complex repeats that are not annotated in UniProt, nor detected as runs of identical amino acid repetitions (see Methods), and thus contribute to the percentage of indels in unique sequence in non-*Hox *genes (Table 3). When complex repeats were annotated and considered as repetitive sequence (see Methods), the percentage of indels in the unique portion of all classes of genes decreased substantially, but especially in non-*Hox *genes [see Additional file 4]. The elimination of complex repeats in cuticular genes was crucial in this reduction, and further increased the differences among groups.

Therefore, our results show that long repetitive sequences are not enough to explain all the differences found between *Hox *or *Hox*-derived genes and non-*Hox *genes. *Hox *and *Hox*-derived genes have a tendency to accumulate indels outside these repetitive regions that is not observed in non-*Hox *genes. We propose that spontaneous deletions between short repeated sequences could be the mechanism responsible for this difference [57]. Such deletions have been described in phages [58,59], *Escherichia coli *[60-65] and humans [66,67], and predominate between short sequence similarities of as few as 5–8 base pairs [68]. Two different models can explain the generation of spontaneous deletions: slipped mispairing during DNA synthesis, and recombination events mediated by enzymes that recognize these sequence similarities. In either case, the repetitive and compositionally biased nature of several regions within *Hox *and *Hox*-derived sequences might explain the major incidence of indels in these two groups. This would also explain the large differences in protein lengths among species that have been observed in some *Hox *proteins [8]. This higher probability of mutation would presumably be accompanied by a higher tolerance to indels of *Hox *and *Hox*-derived proteins outside their binding domains.

For a correct interpretation of our results, the set of non-*Hox *genes should be an unbiased sample of genes, both in terms of protein expression and structure. We have gathered this information from the literature, and verified that our non-*Hox *sample comprises a variable group of genes that are expressed through the fly life cycle (from young embryo to adult) and contains a wide variety of protein domains [see Additional file 5]. Therefore, we assume that, although small, it represents an unbiased sample of all non-*Hox *genes in the genome, and that results presented here are reliable.

### The fate of *Hox*-derived genes after their origination by duplication

The three *Hox*-derived genes used in this study (*bcd*, *zen *and *zen2*) originated from two consecutive duplications of the ancestral *Hox3 *gene. Seemingly, *bcd *and *zen *have specialized and perform separate functions in the establishment of the embryo's body plan [11-13]. This is supported by our data, as these two genes have a moderate evolutionary rate but low level of functional constraint (high *d*_*N*_/*d*_*S *_rate ratio). However, the finding of Barker *et al*. [42] that genes with a maternal effect experience relaxed selective constraint resulting from sex-limited expression is not supported by our data. Our results show that *bcd *and *zen *are evolving at very similar rates in the *Drosophila *lineage, and *bcd *is even more constrained than *zen *[see Additional file 1].

The function of *zen2 *is unclear. It has the same expression pattern as *zen *and, despite its high divergence across species, it has been maintained for more than 60 Myr [19]. Conservation of two paralogous genes maintaining the same function is unlikely, and could only be explained under some peculiar conditions (e.g. two strongly expressed genes whose products are in high demand [40]). It could be that this gene is experiencing a process of pseudogenization, supported by the fact that the evolutionary rate of *zen2 *is more than twice that of *bcd *and *zen*, and that it has also the highest percentage of the alignment represented by indels. If so, we would expect to see a relaxation of the functional constraint. However, the relatively high level of functional constraint of *zen2 *(ω = 0.09144) rather indicates a process of neofunctionalization, even though positive selection was not detected. The fact that this gene does not show an explicit pattern of variation of ω along its sequence (Figure 1) further supports the progressive loss of its original homeotic function and the acquisition of new functions.

Compared to the other two groups (*Hox *and non-*Hox *genes), *Hox*-derived genes are evolving significantly much faster and with less functional constraint. It is also the group with the highest proportion of amino acid differences and indels. These results reflect their relatively recent origin by duplication, which was followed by extensive changes in their role during the development of insects.

## Conclusion

Many studies so far have largely focused on *Hox *gene homeobox sequences, and have demonstrated that they are highly conserved across species. However, *Hox *genes and in general all transcription factors share a particular structure where different highly conserved modules are interspersed with long repetitive regions, mostly microsatellites. Our results show that both *Hox *and *Hox*-derived genes have an overall high rate of evolution, especially in terms of indels. Moreover, although repetitive regions are richer in both amino acid differences and indels than the rest of the coding sequence, they do not seem to fully explain the differences in evolutionary rates found between *Hox *or *Hox*-derived genes and non-*Hox *genes. Therefore, by using complete gene sequences rather than their conserved modules, we observe that the *Hox *gene evolutionary rate is as high as that of non-*Hox *genes in terms of nucleotide evolution, and even higher in terms of indels. *Hox*-derived genes constitute the group with the highest evolutionary rate by all criteria. These results emphasize the need to take into account indels in addition to nucleotide substitutions in order to estimate evolutionary rates accurately. This study is the first quantification of the rates of nucleotide and indel evolution in these groups of genes, and shows that *Hox *and *Hox*-derived genes have a higher evolutionary dynamics than other developmental genes.

## Methods

### Genes analyzed and their classification

All the completely sequenced genes in *D. buzzatii *with a clear ortholog in *D. melanogaster *and *D. pseudoobscura *(23) were included in our analysis: *abd-A*, *lab*, *pb*, *bcd*, *zen*, *zen2*, *Dbuz\Ccp3 *(ortholog of *Dmel\Ccp84Ac*), *Dbuz\Ccp6 *(ortholog of *Dmel\Ccp84Ae*), *Dbuz\Ccp7 *(ortholog of *Dmel\Ccp84Af*), *Dbuz\Ccp8 *(ortholog of *Dmel\Ccp84Ag*), *CG1288*, *CG14290*, *CG14609*, *CG14899*, *CG17836*, *CG2520 *and *CG31363 *from Negre *et al*. [19]; *Adh-related *(*Adhr*) from Betran and Ashburner [69]; *α-Esterase-2 *(*α-Est2*) and *α-Esterase-3 *(*α-Est3*) from Robin *et al*. [70]; *CG13617 *from Puig, Caceres, and Ruiz [71]; and *Larval serum protein 1 β *(*Lsp1β*) and *Lsp1γ *from Gonzalez, Casals and Ruiz [72]. Sequences of *D. melanogaster *orthologs were collected from Flybase [73,74], and those of *D. pseudoobscura *were annotated on the scaffolds from the whole genome shotgun sequencing project [75,76]. We identified the *D. pseudoobscura *orthologs by using the alignment of this species with the *D. melanogaster *genome generated by the Berkeley Genome Pipeline [77], and annotated the target sequences with the aid of ARTEMIS v. 7 [78] and BIOEDIT v. 7.0.4.1 [79]. A complete list of all genes, accession numbers (from Genbank or Flybase) and chromosomal locations is provided [see Additional file 3]. The longest transcript of each gene was used for the analyses. Genes were classified into three categories: 1) *Hox *genes (*abd-A*, *lab *and *pb*); 2) *Hox*-derived genes (*bcd*, *zen *and *zen2*); and 3) non-*Hox *genes (the remaining 17 genes). Results in each group were produced by calculating the average of all the genes within the group.

### Sequence annotation and alignment

A set of Perl scripts, together with modules from PDA v. 1.4 [80] and BIOPERL v. 1.2.3 [81], were used to automatically check sequence annotations, extract the coding sequences (CDSs) of the selected transcripts and calculate basic gene structure and base composition parameters (gene and protein lengths; codon bias measured by the Effective Number of Codons (*N*_*C*_); and G+C content in second, third and all codon positions) [see Additional file 1]. Differences among the three groups of genes were tested with one-way ANOVAs and pairwise contrast tests [82], assuming homogeneity of variances for those variables that gave non-significant P values for the Levene test [83] [see Additional file 2]. Orthologous coding sequences in *D. buzzatii*, *D. melanogaster *and *D. pseudoobscura *were aligned according to their translation to protein using RevTrans 1.3 Server [84] with some manual editing using BIOEDIT v. 7.0.4.1 [79]. Two non-*Hox *genes of the initial sample (*CG1288 *and *CG17836*) showed a doubtful alignment, containing many gaps and few residue matches, and thus were excluded from the analyses to avoid unreliable estimates. A total of 15 non-*Hox *genes were therefore used in this study.

### Estimation of evolutionary rates

The numbers of synonymous and nonsynonymous substitutions per site (*d*_*S *_and *d*_*N*_, respectively) were estimated on the nucleotide alignments of each gene using maximum likelihood methods with the program *codeml *of the PAML v. 3.14 package [85] [see Additional file 1]. We used an unrooted tree and the codon equilibrium frequencies (*π*_*i*_) estimated from the nucleotide frequencies of the three codon sites (F3X4 option of *codeml*). Differences among the three groups of genes were tested using one-way ANOVAs and pairwise contrast tests as before. Furthermore, we visualized differences along the genes by plotting *d*_*N *_and ω in sliding windows of 240 nucleotides and a step size of three nucleotides (one codon).

### Measurement of amino acid differences and indels

We measured the proportion of amino acid differences and indels in the protein alignments (translated from the previous nucleotide alignments) using in-house Perl scripts. The methodology was based on measuring the number of non-conserved positions due to either amino acid differences (point changes) or indels (structural changes) in the protein multiple alignments (e.g. the minimum indel length is one amino acid, corresponding to three nucleotides in the nucleotide sequence). We can estimate in this way the percentage of the protein which has been changed in our set of species. We think that this is a simple (yet somewhat rough) measure to estimate the degree of constraint relaxation of proteins.

Specifically, the number of amino acid differences was computed as the number of non-gapped positions with non-identical amino acids in the three species. All percentages are given in relation to the total number of aligned amino acids (non-gapped positions). Similarly, the number of indels was computed as the number of different indels (gaps affecting different positions) in the complete alignment (gapped and non-gapped sites). Therefore, an indel shared by two species was considered a single indel, while overlapping gaps were considered separately. Indel lengths were taken into account to calculate the percentage of the alignment affected by indels. In this case, all percentages are given in relation to the total length of the alignment (gapped and non-gapped positions).

We used one-way ANOVAs to test for differences between *Hox*, *Hox*-derived and non-*Hox *proteins in both parameters: the proportion of amino acid differences and the proportion of indels. We also used the Pearson correlation coefficient to test for a correlation between the two measures (e.g. to test whether proteins with a high proportion of amino acid differences also have a high proportion of indels), and a nested ANOVA [82] to test for differences in indel length among the three groups, taking into account the variation within groups.

### Contribution of the homeobox and the repetitive regions to the evolutionary rates

In order to test the effect of the homeobox and the repetitive regions in our estimates of nucleotide substitutions, we repeated the previous analyses excluding one or both types of sequence. Repetitive regions were identified in three different ways. First, we searched in the UniProt Knowledgebase Release 8.6 (Swiss-Prot Release 50.6 + TrEMBL Release 33.6) [86] for annotated compositionally biased regions (defined in the feature table as COMPBIAS) in the protein sequences encoded by *Hox*, *Hox*-derived and non-*Hox *genes [see Additional file 3]. In the case of *Hox *genes, all three genes in the group contained at least one annotated repetitive region, while for *Hox*-derived and non-*Hox *genes only one entry of each group (*bcd *and *CG2520*, respectively) contained annotated repetitive regions. Note that only repeats in *D. melanogaster *are identified by using this methodology. Second, we identified simple repeats as those runs of 5 or more identical amino acids (e.g. QQQQQ), or at least 4 identical repetitions of 2 or more amino acids (e.g. GVGVGVGV), in any of the three species. By using this second approach, we extended the number of proteins with repetitive sequences in both the *Hox*-derived and non-*Hox *groups. Finally, we tried to visually annotate complex repeats as those imperfect runs of amino acid repetitions or compositionally biased regions in the protein (e.g. regions in the protein with a high content of Q, S, A, P, H, G, V, etc.). Data was analyzed using a combination of the three approaches as follows: (1) using UniProt only; (2) using UniProt + Simple repeats; and (3) using UniProt + Simple repeats + Complex repeats. Because the identification of complex repeats is somewhat subjective, we present in the main text the results obtained by identifying repeats using the second combination (UniProt + Simple repeats). However, results do not differ significantly among the three combinations [see Additional file 4].

We also calculated the proportion of amino acid differences and indels in repetitive and non-repetitive (unique) sequence in the three groups, and tested for differences between these two types of regions using a T-test for paired samples [82] on those proteins having both types of regions.

## Abbreviations

*t *= number of nucleotide substitutions per codon; *d*_*S *_= number of synonymous substitutions per synonymous site; *d*_*N *_= number of nonsynonymous substitutions per nonsynonymous site; ω = *d*_*N*_/*d*_*S *_ratio that measures the level of functional constraint; *κ *= transition/transversion rate ratio; *N*_*C *_= Effective Number of Codons.

## Authors' contributions

SC carried out the analyses and drafted the manuscript. BN participated in obtaining the data and in the design of the analyses. AB participated in the statistical analysis. AR conceived the study, and participated in its design and coordination. All authors read and approved the final manuscript.

## Supplementary Material

Additional File 1 Parameters of gene structure, base composition and nucleotide evolution for each gene. Click here for file

Additional File 2 ANOVA and contrast analyses for all group comparisons. Click here for file

Additional File 3 Genes from *D. buzzatii*, *D. melanogaster *and *D. pseudoobscura *used in the analyses with their accession number in Genbank or Flybase and their location on the chromosome. Click here for file

Additional File 4 Set of tables of the main text, obtained according to three different annotation criteria to define repetitive sequences. Click here for file

Additional File 5 Structure and expression of non-*Hox *proteins. Click here for file
