# Supplementary material for: Fast sequence evolution of Hox and Hox-derived genes in the genus Drosophila
Source: BMC Evol Biol. 2006 Dec 12;6:106. doi: 10.1186/1471-2148-6-106 (PMC1764764; doi:10.1186/1471-2148-6-106)
Supplement: Additional File 1 — Parameters of gene structure, base composition and nucleotide evolution for each gene. [file 1471-2148-6-106-S1.pdf]

Additional file 1. Parameters of gene structure, base composition and nucleotide evolution for each gene.

Gene	Specie	Gene length[a]	Protein length[b]	C. Bias[c] N_C	G+C content measures[d]			*t*	d_N	d_S	κ	ω
					GC	GC2	GC3					
***Hox* genes**												
abd-A	*Dbuz*	20665	536	51.16	0.5815	0.4795	0.6493	1.82426	0.09071	2.46771	1.66636	0.03676
	Dmel	18315	590	49.63	0.6006	0.4915	0.7080					
	Dpse	18244	568	38.13	0.6450	0.4947	0.8145					
	**Avg**	***19074.7***	***564.7***	***46.31***	***0.6090***	***0.4886***	***0.7240***					
lab	*Dbuz*	21166	659	52.98	0.5544	0.4355	0.5799	2.80665	0.24208	3.28391	1.66998	0.07372
	Dmel	16276	629	41.98	0.6078	0.4674	0.7368					
	Dpse	15388	691	48.00	0.5808	0.4443	0.6642					
	**Avg**	***17610.0***	***659.7***	***47.65***	***0.5810***	***0.4491***	***0.6603***					
pb	*Dbuz*	34287	763	53.81	0.5343	0.4404	0.5670	1.69659	0.14613	2.02037	1.39885	0.07233
	Dmel	31889	782	50.90	0.5631	0.4348	0.6554					
	Dpse	32676	801	50.00	0.5589	0.4444	0.6087					
	**Avg**	***32950.7***	***782.0***	***51.57***	***0.5521***	***0.4399***	***0.6104***					
Hox genes:	*Dbuz*	25372.7	652.7	52.65	0.5567	0.4518	0.5988	**2.10917**	**0.15964**	**2.59066**	**1.57840**	**0.06094**
	Dmel	22160.0	667.0	47.50	0.5905	0.4646	0.7000					
	Dpse	22102.7	686.7	45.38	0.5949	0.4611	0.6958					
	**Avg**	***23211.8***	***668.8***	***48.51***	***0.5807***	***0.4592***	***0.6649***					
***Hox*-derived genes**												
bcd	*Dbuz*	2385	542	52.41	0.5387	0.4428	0.5532	2.37143	0.21122	2.47837	1.70016	0.08523
	Dmel	2593	489	49.76	0.5828	0.4540	0.6624					
	Dpse	1829	536	48.35	0.5777	0.4366	0.6245					
	**Avg**	***2269.0***	***522.3***	***50.17***	***0.5664***	***0.4444***	***0.6134***					
zen	*Dbuz*	1057	331	44.79	0.5498	0.4441	0.6384	2.47931	0.27517	2.74878	1.33133	0.10011
	Dmel	1123	353	48.56	0.5420	0.4448	0.6499					
	Dpse	1198	378	48.76	0.5785	0.4815	0.6458					
	**Avg**	***1126.0***	***354.0***	***47.37***	***0.5568***	***0.4568***	***0.6447***					
zen2	*Dbuz*	958	297	57.00	0.4478	0.4074	0.4134	6.73935	0.69501	7.60078	0.93934	0.09144
	Dmel	823	252	59.77	0.4563	0.3810	0.4669					
	Dpse	880	270	56.94	0.4951	0.3889	0.5625					
	**Avg**	***887.0***	***273.0***	***57.91***	***0.4664***	***0.3924***	***0.4810***					
Hox-derived genes:	*Dbuz*	1466.7	390.0	51.40	0.5121	0.4314	0.5350	**3.86336**	**0.39380**	**4.27598**	**1.32361**	**0.09226**
	Dmel	1513.0	364.7	52.69	0.5271	0.4266	0.5931					
	Dpse	1302.3	394.7	51.35	0.5504	0.4356	0.6109					
	**Avg**	***1427.3***	***383.1***	***51.82***	***0.5299***	***0.4312***	***0.5797***					

Gene	Specie	Gene length[a]	Protein length[b]	C. Bias[c] N_C	G+C content measures[d]			*t*	d_N	d_S	κ	ω
					GC	GC2	GC3					
**Non-*Hox* genes**												
Adhr	*Dbuz*	939	274	45.14	0.5535	0.3650	0.7422	2.49544	0.10802	3.61777	0.95355	0.02986
	Dmel	1293	272	59.09	0.4792	0.3750	0.5333					
	Dpse	1200	278	45.14	0.5396	0.3885	0.6667					
	**Avg**	***1144.0***	***274.7***	***49.79***	***0.5241***	***0.3761***	***0.6474***					
α -Est2	*Dbuz*	1991	565	46.22	0.5558	0.4071	0.6904	3.72951	0.25409	4.94931	1.23819	0.05134
	Dmel	2226	566	56.00	0.5106	0.3710	0.5885					
	Dpse	1978	566	45.14	0.5648	0.3799	0.7234					
	**Avg**	***2065.0***	***565.7***	***49.12***	***0.5437***	***0.3860***	***0.6674***					
α -Est3	*Dbuz*	1820	541	47.73	0.5471	0.4011	0.6842	3.40410	0.25436	4.38895	1.14458	0.05796
	Dmel	2154	543	50.73	0.5476	0.3831	0.6647					
	Dpse	1808	543	44.83	0.5641	0.3923	0.6783					
	**Avg**	***1927.3***	***542.3***	***47.76***	***0.5529***	***0.3921***	***0.6757***					
Ccp84Ac	*Dbuz*	701	215	50.81	0.5705	0.3907	0.6168	5.11649	0.18095	6.70225	1.38891	0.02700
	Dmel	715	217	39.06	0.6129	0.3917	0.7083					
	Dpse	783	231	35.67	0.6075	0.4026	0.6870					
	**Avg**	***733.0***	***221.0***	***41.85***	***0.5970***	***0.3950***	***0.6707***					
Ccp84Ae	*Dbuz*	647	195	45.63	0.5932	0.5026	0.5361	3.87698	0.16555	3.67507	1.16139	0.04505
	Dmel	742	208	38.71	0.6346	0.5144	0.6618					
	Dpse	722	217	43.55	0.6175	0.5161	0.6435					
	**Avg**	***703.7***	***206.7***	***42.63***	***0.6151***	***0.5110***	***0.6138***					
Ccp84Af	*Dbuz*	491	145	36.71	0.6092	0.4000	0.6875	1.98417	0.09926	2.44754	1.68720	0.04055
	Dmel	511	151	35.28	0.6225	0.3907	0.7667					
	Dpse	512	151	40.76	0.6093	0.3775	0.6933					
	**Avg**	***504.7***	***149.0***	***37.59***	***0.6137***	***0.3894***	***0.7158***					
Ccp84Ag	*Dbuz*	573	162	44.07	0.6173	0.4938	0.6584	1.28347	0.04913	1.42169	2.41924	0.03455
	Dmel	788	191	35.13	0.6754	0.5393	0.7526					
	Dpse	690	198	40.37	0.6364	0.5404	0.6244					
	**Avg**	***683.7***	***183.7***	***39.86***	***0.6430***	***0.5245***	***0.6785***					
CG13617	*Dbuz*	2401	734	54.38	0.5050	0.3828	0.5461	4.45418	0.38303	5.12077	1.80499	0.07480
	Dmel	2440	737	50.56	0.5364	0.3894	0.6335					
	Dpse	2420	745	50.60	0.5427	0.3933	0.6598					
	**Avg**	***2420.3***	***738.7***	***51.85***	***0.5280***	***0.3885***	***0.6131***					
CG14290	*Dbuz*	587	108	81.73	0.5278	0.5370	0.5960	4.24639	0.17265	6.25148	0.67744	0.02762
	Dmel	756	107	56.68	0.5483	0.4766	0.6939					
	Dpse	640	107	52.11	0.5701	0.4673	0.7347					
	**Avg**	***661.0***	***107.3***	***63.51***	***0.5487***	***0.4936***	***0.6748***					

Gene	Specie	Gene length[a]	Protein length[b]	C. Bias[c] N_C	G+C content measures[d]			t	d_N	d_S	κ	ω
					GC	GC2	GC3					
CG14609	*Dbuz*	2603	599	51.51	0.4802	0.2838	0.5930	5.31870	0.21768	7.00844	1.87039	0.03106
	Dmel	2191	597	55.15	0.4662	0.2714	0.5986					
	Dpse	2126	594	51.29	0.4703	0.2677	0.5904					
	**Avg**	***2306.7***	***596.7***	***52.65***	***0.4722***	***0.2743***	***0.5940***					
CG14899	*Dbuz*	1023	258	52.22	0.4974	0.3721	0.5667	2.17723	0.08058	2.54854	1.71101	0.03162
	Dmel	923	261	46.42	0.5504	0.3831	0.6911					
	Dpse	982	258	46.08	0.5413	0.3915	0.6502					
	**Avg**	***976.0***	***259.0***	***48.24***	***0.5297***	***0.3822***	***0.6360***					
CG2520	*Dbuz*	14173	477	50.22	0.5059	0.4465	0.5098	0.91112	0.02653	1.04662	2.03996	0.02535
	Dmel	11011	468	56.50	0.5228	0.4423	0.5588					
	Dpse	11681	473	56.62	0.5159	0.4440	0.5439					
	**Avg**	***12288.3***	***472.7***	***54.45***	***0.5149***	***0.4443***	***0.5375***					
CG31363	*Dbuz*	8491	203	57.49	0.4663	0.4877	0.4192	1.95356	0.15912	2.32116	1.30436	0.06855
	Dmel	13033	208	41.71	0.5833	0.4904	0.7376					
	Dpse	12208	227	45.20	0.5786	0.5066	0.7227					
	**Avg**	***11244.0***	***212.7***	***48.13***	***0.5427***	***0.4949***	***0.6265***					
Lsp1β	*Dbuz*	2427	788	39.80	0.4820	0.2605	0.7236	1.18839	0.09959	2.69820	1.62088	0.03691
	Dmel	2435	789	27.24	0.5560	0.2763	0.9260					
	Dpse	2432	787	28.45	0.5417	0.2630	0.8873					
	**Avg**	***2431.3***	***788.0***	***31.83***	***0.5266***	***0.2666***	***0.8456***					
Lsp1γ	*Dbuz*	2376	773	38.94	0.4981	0.2717	0.7348	1.53426	0.11973	2.90247	1.41796	0.04125
	Dmel	2381	772	41.20	0.4965	0.2642	0.7480					
	Dpse	2383	773	29.41	0.5395	0.2600	0.8804					
	**Avg**	***2380.0***	***772.7***	***36.52***	***0.5113***	***0.2653***	***0.7877***					
Non-Hox genes:	*Dbuz*	2749.5	402.5	49.51	0.5340	0.4002	0.6203	**2.91160**	**0.15802**	**3.80668**	**1.49600**	**0.04156**
	Dmel	2906.6	405.8	45.96	0.5562	0.3973	0.6842					
	Dpse	2837.7	409.9	43.68	0.5626	0.3994	0.6924					
	**Avg**	***2831.3***	***406.0***	***46.38***	***0.5509***	***0.3989***	***0.6656***					
ALL CLASSES:	*Dbuz*	5798.1	436.4	50.23	0.5341	0.4120	0.6050	**2.96138**	**0.23715**	**3.55777**	**1.46600**	**0.06492**
	Dmel	5458.0	437.2	47.15	0.5569	0.4111	0.6735					
	Dpse	5370.5	447.2	45.02	0.5655	0.4134	0.6812					
	**Avg**	***5542.2***	***440.3***	***47.46***	***0.5522***	***0.4121***	***0.6532***					

[a] Gene length (in base pairs), excluding 5' and 3' UTRs (includes exons and introns only from the 'start' to the 'stop' codons in the CDS)

[b] Protein length (in amino acids)

[c] Codon Bias measure: *Effective Number of Codons* (Wright 1990)

[d] G+C content measures: percentage of G+C at all coding positions (GC), second coding positions (GC2) and third coding positions (GC3)
