# Supplementary material for: Fast sequence evolution of Hox and Hox-derived genes in the genus Drosophila
Source: BMC Evol Biol. 2006 Dec 12;6:106. doi: 10.1186/1471-2148-6-106 (PMC1764764; doi:10.1186/1471-2148-6-106)
Supplement: Additional File 2 — ANOVA and contrast analyses for all group comparisons. [file 1471-2148-6-106-S2.pdf]

Additional file 2. ANOVA and contrast analyses for all group comparisons. Numbers refer to tests significances, and those in red are significant (P<0.05). Contrast tests assume homogeneity of variances for all variables except for "Gene length", "Protein length", "*t*" and "d_N" (which gave significant P values for the Levene test).

Pairwise comparisons	Gene length[a]	Protein length[b]	C. Bias[c] N_C	G+C content measures[d]			*t*	d_N	d_S	κ	ω
				GC	GC2	GC3					
**ANOVA**	**0.000**	0.183	0.508	0.409	0.427	0.215	0.404	**0.022**	0.530	0.742	**0.000**
***Hox* ↔ *Hox*-derived**	**0.046**	**0.043**	0.592	0.191	0.658	0.182	0.347	0.259	0.297	0.465	**0.023**
Hox* ↔ Non-*Hox	**0.048**	**0.021**	0.656	0.318	0.227	0.988	0.161	0.976	0.330	0.759	0.063
Hox*-derived ↔ Non-*Hox	0.199	0.821	0.263	0.477	0.511	0.087	0.581	0.259	0.704	0.522	**0.000**

[a] Gene length (in base pairs), excluding 5' and 3' UTRs (includes exons and introns only from the 'start' to the 'stop' codons in the CDS)

[b] Protein length (in amino acids)

[c] Codon Bias measure: *Effective Number of Codons* (Wright 1990)

[d] G+C content measures: percentage of G+C at all coding positions (GC), second coding positions (GC2) and third coding positions (GC3)
