# Supplementary material for: Fast sequence evolution of Hox and Hox-derived genes in the genus Drosophila
Source: BMC Evol Biol. 2006 Dec 12;6:106. doi: 10.1186/1471-2148-6-106 (PMC1764764; doi:10.1186/1471-2148-6-106)
Supplement: Additional File 3 — Genes from D. buzzatii, D. melanogaster and D. pseudoobscura used in the analyses with their accession number in Genbank or Flybase and their location on the chromosome. [file 1471-2148-6-106-S3.pdf]

Additional file 3. Genes from *D. buzzatii* (*Dbuz*), *D. melanogaster* (*Dmel*) and *D. pseudoobscura* (*Dpse*) used in the analyses with their accession number in Genbank or Flybase and their location on the chromosome. In the case of *D. buzzatii*, the original references where the sequences were first published are also supplied. The last column contains the UniProt Knowledgebase accession numbers used for annotating repetitive blocks.

Gene symbol	Tr[a]	Tr used[b]	Intron size[c]	*Dbuz*			*Dmel*		*Dpse*		UniProt Knowledge base acc.
				Genbank acc.	Location	Reference	Flybase acc.	Location	Genbank acc.	Location	
**Hox genes**											
abd-A	2	PB	17448	AY900631	*2, G4g*	Negre et al. 2005	FBgn0000014	*3R, 89E2*	AADE01000036	*2, Cont1892*	P29555
lab	1	PA	14389	AY900631	*2, G4g-5a*	Negre et al. 2005	FBgn0002522	*3R, 84A1*	AADE01000437	*2, Cont1676*	P10105
pb	4	PA	30536	AY900632	*2, F1c*	Negre et al. 2005	FBgn0051481	*3R, 84A5*	AADE01000149	*2, Cont1677*	P31264
**Hox-derived genes**											
bcd	3	PD	1126	AY900632	*2, F1c*	Negre et al. 2005	FBgn0000166	*3R, 84A5*	AADE01000149	*2, Cont1677*	P09081
zen	1	PA	64	AY900632	*2, F1c*	Negre et al. 2005	FBgn0004053	*3R, 84A5*	AADE01000149	*2, Cont1677*	P09089
zen2	1	PA	67	AY900632	*2, F1c*	Negre et al. 2005	FBgn0004054	*3R, 84A5*	AADE01000149	*2, Cont1677*	P09090
**Non-Hox genes**											
Adhr	1	PA	0	AF260699	*3, G1a*	Betran and Ashburner 2000	FBgn0000056	*2L, 35B3*	AADE01001152	*4, Cont1559*	P91615
α *-Est2*	1	PA	528	AF216210	*2, F5e-6a*	Robin et al. 2000	FBgn0015570	*3R, 84D9*	AADE01001585	*2, Cont390*	Q961N0
α *-Est3*	1	PA	835	AF216211	*2, F5e-6a*	Robin et al. 2000	FBgn0015571	*3R, 84D9*	AADE01001585	*2, Cont390*	Q8SZW5
Ccp3 – Ccp84Ac	1	PA	64	AY900631	*2, G4g*	Negre et al. 2005	FBgn0004781	*3R, 84A3--4*	AADE01000149	*2, Cont1677*	O97060
Ccp6 – Ccp84Ae	1	PA	118	AY900631	*2, G4g*	Negre et al. 2005	FBgn0004779	*3R, 84A3*	AADE01000437	*2, Cont1676*	O97062
Ccp7 – Ccp84Af	1	PA	58	AY900631	*2, G4g*	Negre et al. 2005	FBgn0004778	*3R, 84A3*	AADE01000437	*2, Cont1676*	O97063
Ccp8 – Ccp84Ag	1	PA	215	AY900631	*2, G4g*	Negre et al. 2005	FBgn0004777	*3R, 84A2*	AADE01000437	*2, Cont1676*	O97064
CG1288	1	PA	55	AY900632	*2, F1c*	Negre et al. 2005	FBgn0037484	*3R, 84D2*	AADE01000014	*2, Cont268*	Q9VI77
CG13617	1	PA	229	AY551073	*2, E4g-5a*	Puig, Caceres, and Ruiz 2004	FBgn0039201	*3R, 96A5*	AADE01000267	*2, Cont3813*	Q9VC70
CG14290	1	PB	509	AY900632	*2, F1c*	Negre et al. 2005	FBgn0038662	*3R, 91D5*	AADE01000175	*4, Cont2631*	Q7KSC4
CG14609	1	PA	400	AY900632	*2, F1c*	Negre et al. 2005	FBgn0037483	*3R, 84D2*	AADE01000014	*2, Cont268*	Q8T3W3
CG14899	1	PA	140	AY900632	*2, F1c*	Negre et al. 2005	FBgn0038438	*3R, 89B18*	AADE01000429	*2, Cont2827*	Q9VEU2
CG17836	4	PA	5563	AY900632	*2, F1c*	Negre et al. 2005	FBgn0038661	*3R, 91D4--5*	AADE01000175<br>AADE01000328	*4, Cont2631*<br>*4, Cont3752*	Q9VE23
CG2520	1	PA	9607	AY900632	*2, F1c*	Negre et al. 2005	FBgn0026210	*3R, 84D1--2*	AADE01000014	*2, Cont268*	Q9VI75
CG31363	4	PE	12711	AY900631	*2, G4g*	Negre et al. 2005	FBgn0051363	*3R, 86E11--13*	AADE01002495<br>AADE01000322<br>AADE01006966<br>AADE01000546	*2, Cont1082*<br>*2, Cont2641*<br>*2, Cont5794*<br>*2, Cont1019*	Q9VGL6
Lsp1 β	1	PA	68	AY561258	*2, D3c*	Gonzalez, Casals, and Ruiz 2004	FBgn0002563	*2L, 21E2*	AADE01000478	*4, Cont3520*	Q6Q431
Lsp1 γ	1	PA	65	AY561259	*3, C2g*	Gonzalez, Casals, and Ruiz 2004	FBgn0002564	*3L, 61A6*	AADE01000061	*4, Cont1620*	Q6Q429

[a] Number of transcripts that exist in all the three species.

[b] The longest transcript shared by all the three species and that was used for the analyses.

[c] Total intron size in base pairs (sum of all introns in the gene, including those outside the limits of the coding sequence, in*D. melanogaster*).

<100 <200 <500 <1000 >1000
