# Supplementary material for: Fast sequence evolution of Hox and Hox-derived genes in the genus Drosophila
Source: BMC Evol Biol. 2006 Dec 12;6:106. doi: 10.1186/1471-2148-6-106 (PMC1764764; doi:10.1186/1471-2148-6-106)
Supplement: Additional File 4 — Set of tables of the main text, obtained according to three different annotation criteria to define repetitive sequences. [file 1471-2148-6-106-S4.pdf]

Additional file 4:

This file contains Tables 1, 2 and 3 of the main text, obtained according to three different annotation criteria to define repetitive sequences (see ***Methods*** in the main text):

- **Annotation criterion 1 = Uniprot**
- **Annotation criterion 2 = Uniprot + Simple repeats** (note that this is the annotation criterion selected for the main text; tables in this section are those shown in the main text)
- **Annotation criterion 3 = Uniprot + Simple repeats + Complex repeats**

ANNOTATION CRITERION 1 = Uniprot

Table 1. Mean nucleotide substitution parameters, ANOVAs and contrast tests for the three groups of genes.

		t	d_N	d_S	ω
Complete coding sequences	*Hox*	2.10917	0.15964	2.59066	0.06094 ┐ *
	Hox-derived	3.86337	0.39380	4.27598	0.09226 ┤ ***
	Non-*Hox*	2.91160	0.15802	3.80668	0.04156 ┘
	ANOVA	n.s.	*	n.s.	***
Coding sequences excluding the homeobox	*Hox*	2.27653	0.18257	2.65921	0.06673 ┐ ** ┐
	Hox-derived	5.04914	0.54809	5.26666	0.11320 ┤ *** │ *
	Non-*Hox*	2.91160	0.15802	3.80668	0.04156 ┘ ┘
	ANOVA	n.s.	**	n.s.	***
Coding sequences excluding repetitive regions	*Hox*	1.88711 ┐	0.13826	2.36833	0.05917 ┐ *
	Hox-derived	3.80869 │ *	0.39291	4.21256	0.09423 ┤ ***
	Non-*Hox*	2.91375 ┘	0.15726	3.81781	0.04071 ┘
	ANOVA	n.s.	*	n.s.	***
Coding sequences excluding the homeobox and repetitive regions	*Hox*	2.01610	0.16274	2.35658 ┐	0.06782 ┐ **
	Hox-derived	4.99467	0.54854	5.20047 │ *	0.11617 ┤ ***
	Non-*Hox*	2.91375	0.15726	3.81781 ┘	0.04071 ┘
	ANOVA	n.s.	**	n.s.	***

n.s. (P>0.05), * (P<0.05), ** (P<0.01), *** (P<0.001). For pairwise comparisons, only significant values are shown. Contrast tests assume homogeneity of variances where the Levene test does not give a significant P value.

Table 2. Percentage of amino acid differences in the alignment (±SD) in the three groups of proteins.

	TOTAL	UNIQUE	REPETITIVE	T-test§
Hox	22.80 ± *10.44*	19.88 ± *10.84*	33.94 ± *11.71*	
***Hox*-derived**	40.43 ± *18.26*	40.17 ± *18.52*	33.93	**
**Non-*Hox***	23.77 ± *10.81*	23.66 ± *11.02*	13.51	
**ANOVA**	n.s.	n.s.	n.s.	

n.s. (P>0.05), * (P<0.05), ** (P<0.01), *** (P<0.001)

§ T-test for paired samples (unique *vs.* repetitive) on proteins having both types of regions [ABD-A, LAB, PB, BCD and LAP (product of *CG2520*)].

Table 3. Percentage of indels in the alignment (±SD) in the three groups of proteins.

	TOTAL	UNIQUE	REPETITIVE	T-test§
Hox	25.77 ± *4.31*	21.61 ± *6.67*	35.97 ± *5.52*	
***Hox*-derived**	37.53 ± *9.63*	35.36 ± *12.80*	60.61	*
**Non-*Hox***	8.73 ± *10.24*	8.50 ± *10.34*	20.88	
**ANOVA**	***	**	n.s.	

n.s. (P>0.05), * (P<0.05), ** (P<0.01), *** (P<0.001)

§ T-test for paired samples (unique *vs.* repetitive) on proteins having both types of regions [ABD-A, LAB, PB, BCD and LAP (product of *CG2520*)].

ANNOTATION CRITERION 2 = Uniprot + Simple repeats

Note that this is the annotation criterion selected for the main text (tables in this section are those shown in the main text).

Table 1. Mean nucleotide substitution parameters, ANOVAs and contrast tests for the three groups of genes.

		t		d_N	d_S		ω		
Complete coding sequences	*Hox*	2.10917		0.15964	2.59066		0.06094	* (*Hox* vs *Hox*-derived)	
	Hox-derived	3.86336		0.39380	4.27598		0.09226	*** (*Hox*-derived vs Non-*Hox*)	
	Non-*Hox*	2.91160		0.15802	3.80668		0.04156		
	ANOVA	n.s.		*	n.s.		***		
Coding sequences excluding the homeobox	*Hox*	2.27653		0.18257	2.65921		0.06673	** (*Hox* vs *Hox*-derived)	* (*Hox* vs Non-*Hox*)
	Hox-derived	5.04914		0.54809	5.26666		0.11320	*** (*Hox*-derived vs Non-*Hox*)	
	Non-*Hox*	2.91160		0.15802	3.80668		0.04156		
	ANOVA	n.s.		**	n.s.		***		
Coding sequences excluding repetitive regions	*Hox*	1.81997	* (*Hox* vs Non-*Hox*)	0.12399	2.35029	* (*Hox* vs Non-*Hox*)	0.05310	* (*Hox* vs *Hox*-derived)	
	Hox-derived	3.71981		0.37759	4.14242		0.09042	*** (*Hox*-derived vs Non-*Hox*)	
	Non-*Hox*	2.85593		0.15444	3.76458		0.04035		
	ANOVA	n.s.		*	n.s.		***		
Coding sequences excluding the homeobox and repetitive regions	*Hox*	1.94286		0.14684	2.33783	** (*Hox* vs Non-*Hox*)	0.06146	** (*Hox* vs *Hox*-derived)	
	Hox-derived	4.88928		0.53011	5.12014		0.11245	*** (*Hox*-derived vs Non-*Hox*)	
	Non-*Hox*	2.85593		0.15444	3.76458		0.04035		
	ANOVA	n.s.		**	n.s.		***		

n.s. (P>0.05), * (P<0.05), ** (P<0.01), *** (P<0.001). For pairwise comparisons, only significant values are shown. Contrast tests assume homogeneity of variances where the Levene test does not give a significant P value.

Table 2. Percentage of amino acid differences in the alignment (±SD) in the three groups of proteins.

	TOTAL	UNIQUE	REPETITIVE	T-test[§]
Hox	22.80 ± *10.44*	18.22 ± *10.50*	37.11 ± *12.33*	
***Hox*-derived**	40.43 ± *18.26*	39.00 ± *19.64*	62.97 ± *24.08*	***
**Non-*Hox***	23.77 ± *10.81*	23.38 ± *10.93*	55.46 ± *31.35*	
**ANOVA**	n.s.	n.s.	n.s.	

n.s. (P>0.05), * (P<0.05), ** (P<0.01), *** (P<0.001)

[§] T-test for paired samples (unique *vs.* repetitive) on proteins having both types of regions [ABD-A, LAB, PB, BCD, ZEN, Ccp84Ac, CG13617, CG14290 and LAP (product of *CG2520*)].

Table 3. Percentage of indels in the alignment (±SD) in the three groups of proteins.

	TOTAL	UNIQUE	REPETITIVE	T-test[§]
Hox	25.77 ± *4.31*	16.21 ± *8.40*	44.82 ± *2.38*	
***Hox*-derived**	37.53 ± *9.63*	34.88 ± *12.40*	75.64 ± *34.45*	**
**Non-*Hox***	8.73 ± *10.24*	8.46 ± *10.28*	23.79 ± *25.66*	
**ANOVA**	***	**	n.s.	

n.s. (P>0.05), * (P<0.05), ** (P<0.01), *** (P<0.001)

[§] T-test for paired samples (unique *vs.* repetitive) on proteins having both types of regions [ABD-A, LAB, PB, BCD, ZEN, Ccp84Ac, CG13617, CG14290 and LAP (product of *CG2520*)].

ANNOTATION CRITERION 3 = Uniprot + Simple repeats + Complex repeats

Table 1. Mean nucleotide substitution parameters, ANOVAs and contrast tests for the three groups of genes.

		t		d_N	d_S		ω		
Complete coding sequences	*Hox*	2.10917		0.15964	2.59066		0.06094	*	
	Hox-derived	3.86337		0.39380	4.27598		0.09226		
	Non-*Hox*	2.91160		0.15802	3.80668		0.04156	***	
	ANOVA	n.s.		*	n.s.		***		
Coding sequences excluding the homeobox	*Hox*	2.27653		0.18257	2.65921		0.06673	**	
	Hox-derived	5.04914		0.54809	5.26666		0.11320		*
	Non-*Hox*	2.91160		0.15802	3.80668		0.04156	***	
	ANOVA	n.s.		**	n.s.		***		
Coding sequences excluding repetitive regions	*Hox*	1.47343		0.08116	2.09576		0.03615	*	
	Hox-derived	3.57569	*	0.33831	4.17038	*	0.07474		
	Non-*Hox*	2.69427		0.14241	3.72116		0.03739	**	
	ANOVA	n.s.		n.s.	n.s.		*		
Coding sequences excluding the homeobox and repetitive regions	*Hox*	1.52473		0.09941	2.00530		0.04554		
	Hox-derived	4.70398	*	0.48080	5.11828	**	0.09217		
	Non-*Hox*	2.69427		0.14241	3.72116		0.03739	***	
	ANOVA	n.s.		*	n.s.		**		

n.s. (P>0.05), * (P<0.05), ** (P<0.01), *** (P<0.001). For pairwise comparisons, only significant values are shown. Contrast tests assume homogeneity of variances where the Levene test does not give a significant P value.

Table 2. Percentage of amino acid differences in the alignment (±SD) in the three groups of proteins.

	TOTAL	UNIQUE	REPETITIVE	T-test§
Hox	22.80 ± *10.44*	13.64 ± *11.65*	37.00 ± *10.69*	
***Hox*-derived**	40.43 ± *18.26*	35.87 ± *22.13*	57.50 ± *9.77*	**
**Non-*Hox***	23.77 ± *10.81*	21.75 ± *11.57*	34.16 ± *31.78*	
**ANOVA**	n.s.	n.s.	n.s.	

n.s. (P>0.05), * (P<0.05), ** (P<0.01), *** (P<0.001)

§ T-test for paired samples (unique *vs.* repetitive) on proteins having both types of regions [ABD-A, LAB, PB, BCD, ZEN, Ccp84Ac, Ccp84Ae, Ccp84Af, Ccp84Ag, CG13617, CG14290, LAP (product of *CG2520*) and CG31363].

Table 3. Percentage of indels in the alignment (±SD) in the three groups of proteins.

	TOTAL	UNIQUE	REPETITIVE	T-test§
Hox	25.77 ± *4.31*	9.72 ± *5.29*	43.60 ± *6.39*	
***Hox*-derived**	37.53 ± *9.63*	27.78 ± *17.38*	66.43 ± *7.83*	***
**Non-*Hox***	8.73 ± *10.24*	3.45 ± *5.29*	23.41 ± *20.84*	
**ANOVA**	***	***	*	

n.s. (P>0.05), * (P<0.05), ** (P<0.01), *** (P<0.001)

§ T-test for paired samples (unique *vs.* repetitive) on proteins having both types of regions [ABD-A, LAB, PB, BCD, ZEN, Ccp84Ac, Ccp84Ae, Ccp84Af, Ccp84Ag, CG13617, CG14290, LAP (product of *CG2520*) and CG31363].
