# Supplementary material for: Fast sequence evolution of Hox and Hox-derived genes in the genus Drosophila
Source: BMC Evol Biol. 2006 Dec 12;6:106. doi: 10.1186/1471-2148-6-106 (PMC1764764; doi:10.1186/1471-2148-6-106)
Supplement: Additional File 5 — Structure and expression of non-Hox proteins. [file 1471-2148-6-106-S5.pdf]

Additional file 5. Structure and expression of non-*Hox* proteins.

Gene symbol	Protein expression	Protein structure
Adhr	Enzyme with oxidoreductase activity that is expressed always with ADH in the embryo gastric caecae, larvae and the adult fat body and gut *[1]*.	It contains a short chain dehydrogenase domain (adh-short; PF00106).
α-Est2	Enzymes that act on carboxylic esters during embryogenesis, larvae and pupae *[2]*.	The catalytic apparatus (Carboxylesterase; PF00135) involves three residues (the catalytic triad): a serine, a glutamate or aspartate, and a histidine.
α-Est3		
Ccp3 – Ccp84Ac	Structural constituent of the larval cuticle.	Conserved C-terminal section *[3]* and include a 35-36 amino acid motif known as the R&R consensus, present in many insect cuticle proteins, an extended form of which has been shown to bind chitin (chitin-bind 4; PF00379) *[4]*. Outside these conserved domains, cuticular proteins share hydrophobic regions dominated by tetrapeptide repeats (A-A-P-A/V), which are presumed to be functionally important *[5,6]* and are responsible for the high percentage of indels found in these proteins.
Ccp6 – Ccp84Ae		
Ccp7 – Ccp84Af		
Ccp8 – Ccp84Ag		
CG13617	Nucleic acid/zinc ion binding protein.	It contains a classical zinc finger domain (zf-C2H2; PF00096). The C2H2 zinc finger is composed of 25 to 30 amino acid residues including 2 conserved Cys and 2 conserved His residues in a C-2-C-12-H-3-H type motif. The 12 residues separating the second Cys and the first His are mainly polar and basic, implicating this region in particular in nucleic acid binding. The zinc finger motif is an unusually small, self-folding domain in which Zn is a crucial component of its tertiary structure binding to the conserved Cys and His residues. Fingers have been found to bind to about 5 base pairs of nucleic acid containing short runs of guanine residues.
CG14290	Unknown	No domains identified.
CG14609	Unknown	No domains identified.
CG14899	Could be a membrane protein.	Putative membrane domains predicted.
CG2520	Binding protein that is expressed in the embryonic nervous system and garland cell, and in the larval neuromuscular synapses.	It contains three defined domains: an ENTH domain of unknown function (PF01417) located at the N-termini and composed of 9 alpha-helices connected by loops; a phosphoinositide-binding clathrin adaptor (IPR008943) involved in clathrin-mediated endocytosis; and an ANTH domain (PF07651) involved in phosphatidylinositol 4,5-bisphosphate binding. It contains annotated repetitive sequences in UniProt.
CG31363	Soluble unfolded molecule associated with microtubules through the cell cycle *[7]*. It is expressed in the young embryo, larval nervous system, precursors of eye photoreceptors and adult ovary.	No domains identified. It contains 2 degenerated repeats around the sequence PPGG, separated by a Serine-rich region *[7]*.
Lsp1β	Proteins from the hemolymph of insects, which are expressed in larvae and may serve as a store of amino acids for synthesis of adult proteins.	Structurally related to arthropod hemocyanins. They contain an N-terminal domain (Hemocyanin-N; PF03722), a copper-containing domain (Hemocyanin-M; PF00372) and a C-terminal ig-like domain (Hemocyanin-C; PF03723).
Lsp1γ		

References:

1. Betran E, Ashburner M: **Duplication, dicistronic transcription, and subsequent evolution of the Alcohol dehydrogenase and Alcohol dehydrogenase-related genes in Drosophila.** *Mol Biol Evol* 2000, **17:**1344-1352.

2. Arbeitman MN, Furlong EE, Imam F, Johnson E, Null BH, Baker BS, Krasnow MA, Scott MP, Davis RW, White KP: **Gene expression during the life cycle of Drosophila melanogaster.** *Science* 2002, **297:**2270-2275.

3. Rebers JE, Riddiford LM: **Structure and expression of a Manduca sexta larval cuticle gene homologous to Drosophila cuticle genes.** *J Mol Biol* 1988, **203:**411-423.

4. Rebers JE, Willis JH: **A conserved domain in arthropod cuticular proteins binds chitin.** *Insect Biochem Mol Biol* 2001, **31:**1083-1093.

5. Talbo G, Hojrup P, Rahbek-Nielsen H, Andersen SO, Roepstorff P: **Determination of the covalent structure of an N- and C-terminally blocked glycoprotein from endocuticle of Locusta migratoria. Combined use of plasma desorption mass spectrometry and Edman degradation to study post-translationally modified proteins.** *Eur J Biochem* 1991, **195:**495-504.

6. Andersen SO, Rafn K, Roepstorff P: **Sequence studies of proteins from larval and pupal cuticle of the yellow meal worm, Tenebrio molitor.** *Insect Biochem Mol Biol* 1997, **27:**121-131.

7. Karpova N, Bobinnec Y, Fouix S, Huitorel P, Debec A: **Jupiter, a new Drosophila protein associated with microtubules.** *Cell Motil Cytoskeleton* 2006, **63:**301-312.
